# The association between latent trauma and brain structure in children

**DOI:** 10.1038/s41398-021-01357-z

**Published:** 2021-04-24

**Authors:** Hee Jung Jeong, E. Leighton Durham, Tyler M. Moore, Randolph M. Dupont, Malerie McDowell, Carlos Cardenas-Iniguez, Emily T. Micciche, Marc G. Berman, Benjamin B. Lahey, Antonia N. Kaczkurkin

**Affiliations:** 1 Department of Psychology, Vanderbilt University, Nashville, TN USA; 2 Department of Psychiatry, Perelman School of Medicine, University of Pennsylvania, Philadelphia, PA USA; 3 Department of Psychology, University of Chicago, Chicago, IL USA; 4 The Grossman Institute for Neuroscience, Quantitative Biology and Human Behavior, University of Chicago, Chicago, IL USA; 5 Departments of Health Studies and Psychiatry and Behavioral Neuroscience, University of Chicago, Chicago, IL USA

**Keywords:** Neuroscience, Psychology

## Abstract

The developing brain is marked by high plasticity, which can lead to vulnerability to early life stressors. Previous studies indicate that childhood maltreatment is associated with structural aberrations across a number of brain regions. However, prior work is limited by small sample sizes, heterogeneous age groups, the examination of one structure in isolation, the confounding of different types of early life stressors, and not accounting for socioeconomic status. These limitations may contribute to high variability across studies. The present study aimed to investigate how trauma is specifically associated with cortical thickness and gray matter volume (GMV) differences by leveraging a large sample of children (*N* = 9270) from the Adolescent Brain Cognitive Development^SM^ Study (ABCD Study^®^). A latent measure of trauma exposure was derived from *DSM-5* traumatic events, and we related this measure of trauma to the brain using structural equation modeling. Trauma exposure was associated with thinner cortices in the bilateral superior frontal gyri and right caudal middle frontal gyrus (*p*_fdr_*-*values < .001) as well as thicker cortices in the left isthmus cingulate and posterior cingulate (*p*_fdr_*-*values ≤ .027), after controlling age, sex, and race/ethnicity. Furthermore, trauma exposure was associated with smaller GMV in the right amygdala and right putamen (*p*_fdr_*-*values ≤ .048). Sensitivity analyses that controlled for income and parental education were largely consistent with the main findings for cortical thickness. These results suggest that trauma may be an important risk factor for structural aberrations, specifically for cortical thickness differences in frontal and cingulate regions in children.

## Introduction

Childhood is a sensitive period marked by high brain plasticity^1^. The developing brain undergoes substantial change during this time, including simultaneous neuronal proliferation and pruning as well as considerable rewiring of existing neuronal connections^2^. While plasticity may be adaptive, as in the case of recovery from brain injury, such malleability also suggests the potential for chronic adverse events such as childhood maltreatment to have a greater impact during this sensitive period. Thus, it is important to understand the effect of adverse early life experiences on structural brain development.

Childhood maltreatment is a broad term that often includes early life stress, neglect, emotional ill-treatment, and trauma. A growing body of literature suggests that childhood maltreatment is associated with aberrations in brain structures measured with volume and cortical thickness including the prefrontal cortex^3–8^, cingulate cortex^9–13^, limbic structures^7,9,12,14–19^, and temporal lobe^3,7,15,20,21^. However, there is substantial variability across these studies, with some studies showing smaller volumes or thinner cortices in these regions, while others find greater volume or thicker cortices. Crucially, the particular regions associated with childhood maltreatment are not consistent across studies. In general, childhood maltreatment appears to be associated with smaller brain volume, with studies finding smaller volumes in divergent regions such as various frontal, temporal and parietal regions, anterior cingulate cortex, hippocampus/parahippocampal gyri, lingual gyrus, amygdala, insula, caudate, precuneus, and cerebellum^8,9,12–17,20–23^, while others find larger volumes in some of these same regions^4,19,21,24^. Childhood maltreatment is also associated with cortical thinning, with thinner cortices found in frontal and temporal regions, parahippocampal gyri, anterior and posterior cingulate cortices, and the paracentral lobule^3,7,10,11,21^, although thicker cortices also have been reported for the posterior cingulate cortex and paracentral lobule^24^.

There may be a number of reasons for the inconsistent results found in previous studies. First, most prior work has been limited by small sample sizes, which impacts the power to detect effects and the reproducibility of results. Second, previous studies have used samples with broad age ranges in which the association with trauma may be masked by region-specific developmental change. Third, studies on childhood maltreatment often examine one structural measure, such as volume or cortical thickness, in isolation^3–6,8,10,12,14,19,20,22,23,25^ despite evidence of structural abnormalities in both^21,24^, which would allow investigators to determine the correspondence or divergence in results between these measures. Fourth, prior work often lacks controls for measures of socioeconomic status (SES), which are known to have associations with the development of brain structures^26^ and also early life trauma^27^. Fifth, and perhaps most importantly, many studies confound diverse early life experiences such as stress, neglect, emotional ill-treatment, and trauma exposure, which are aggregated into the broad concept of childhood maltreatment. This confounding makes it difficult to disentangle the relative contribution of each early-life experience, and may contribute to the high degree of variability found across studies.

Of the studies that examine trauma exposure specifically, most are limited to post-traumatic stress disorder (PTSD) symptom severity^6,16,17,24,25^ or case–control studies comparing those with a diagnosis of PTSD to healthy controls^3,4,6,14,16,17^. Focusing on those with PTSD overlooks the large number of children who are exposed to trauma but do not go on to develop PTSD. A study based on a national sample indicates the percentage of children who experienced at least one traumatic event under age 9 was 40.5%^28^. While not all individuals who are exposed to trauma develop PTSD^29^, studies indicate that childhood trauma is associated with non-specific mental illnesses^30^, psychosocial problems^31^, and health-related problems^32^. The high prevalence and increased risk of a wide range of problems associated with childhood trauma highlight the importance of investigating trauma exposure in children.

A latent measure of trauma may be useful for creating an index of trauma exposure that minimizes measurement error. Compared to a simple count of traumatic events, the main benefit of a latent variable is that it accounts for communalities and measurement error by weighting trauma events based on how well they predict each other^33^. The noise introduced by trauma events that do not predict the other events well will be minimized, since that item will have a smaller weight than the more predictive trauma items. While the correlation between a simple count score and latent trait score is very high, the current study uses a latent measure of trauma exposure to reduce this extraneous measurement error.

The current study builds upon prior work in several important ways. To overcome the limitation of small sample sizes, we utilized data from 9270 children from the first wave of the Adolescent Brain Cognitive Development (ABCD) Study^34^. This sample is restricted to children between 9 and 10 years of age, which allows us to avoid confounding across broad age ranges. Additionally, we examined both cortical thickness and gray matter volume (GMV) in the same sample, allowing for the comparison of results across structural modalities. We also included parental education and income to control for the confounding effects of SES. Finally, we extend prior work by deriving a latent variable of trauma based on *Diagnostic and Statistical Manual of Mental Disorders* (*DSM-5*) traumatic events to understand the effects of trauma exposure specifically on brain structure. We hypothesized that our latent trauma variable, which measures exposure to a greater number of traumatic events while accounting for measurement error, would be associated with smaller volumes and thinner cortices. Given the variability in regions found across prior studies, we did not have an a priori prediction about whether these associations would be global or focal in the current study.

## Methods

### Participants

The present study used data from Wave 1 of the ABCD Study (release 2.0.1), which includes de-identified data and curated imaging data from 11,875 children between the ages of 9 and 10 years^34^. The initial sample was collected at 21 sites distributed across the United States^35^. Post-stratification weights were used to adjust the sample to be more representative of the US population^36^. Parental consent and children’s assent were obtained by the ABCD group. For the current study, the institutional review board of Vanderbilt University approved the use of this deidentified dataset. The final sample size was *N* = 9270 following the exclusion of missing data and participants failing to pass quality assurance measures (Supplementary Fig. 1). A summary of demographics based on the final sample can be found in Table 1.

**Table 1:** Demographics of the sample (*N* = 9270).

			Mean	SD
Age (months)			119.17	7.47
			*N*	%
*Gender*				
Female			4519	48.75
Male			4751	51.25
*Race/Ethnicity*				
White			4956	53.46
Hispanic			1860	20.06
African American			1329	14.34
Other			1125	12.14
*Household annual income*				
<$5000			311	3.35
$5000–$11,999			310	3.34
$12,000-$15,999			215	2.32
$16,000–$24,999			392	4.23
$25,000–$34,999			501	5.40
$35 000-$49 999			708	7.64
$50 000-$74 999			1164	12.56
$75 000-$99 999			1249	13.47
$100 000-$199 999			2664	28.74
> $200 000			986	10.64
Missing			770	8.31
*Parental education*				
No degree			453	4.89
High school degree/GED			1104	11.91
Some college			1499	16.17
Associate’s degree			1174	12.66
Bachelor’s degree			2626	28.33
Master’s degree			1824	19.68
Professional/Doctoral degree			577	6.22
Missing			13	0.14

The “Other” Race/Ethnicity category includes those who were identified by their parent as American Indian/Native American, Alaska Native, Native Hawaiian, Guamanian, Samoan, Other Pacific Islander, Asian Indian, Chinese, Filipino, Japanese, Korean, Vietnamese, Other Asian, or Other Race.

### Trauma measure

**Fig. 1:**
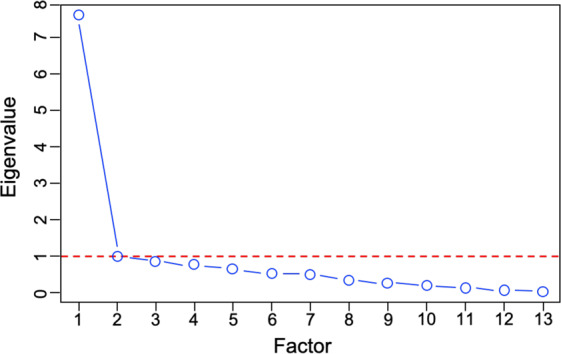
Exploratory factor analysis identifies a single trauma exposure factor. Exploratory factor analysis was conducted with 13 trauma items. The number of factors is plotted along the *x*-axis while the eigenvalues are plotted along the *y*-axis. The screen plot of the results indicates a steep decrease (“elbow”) at two factors, suggesting that the trauma items are represented by single factor.

**Fig. 2:**
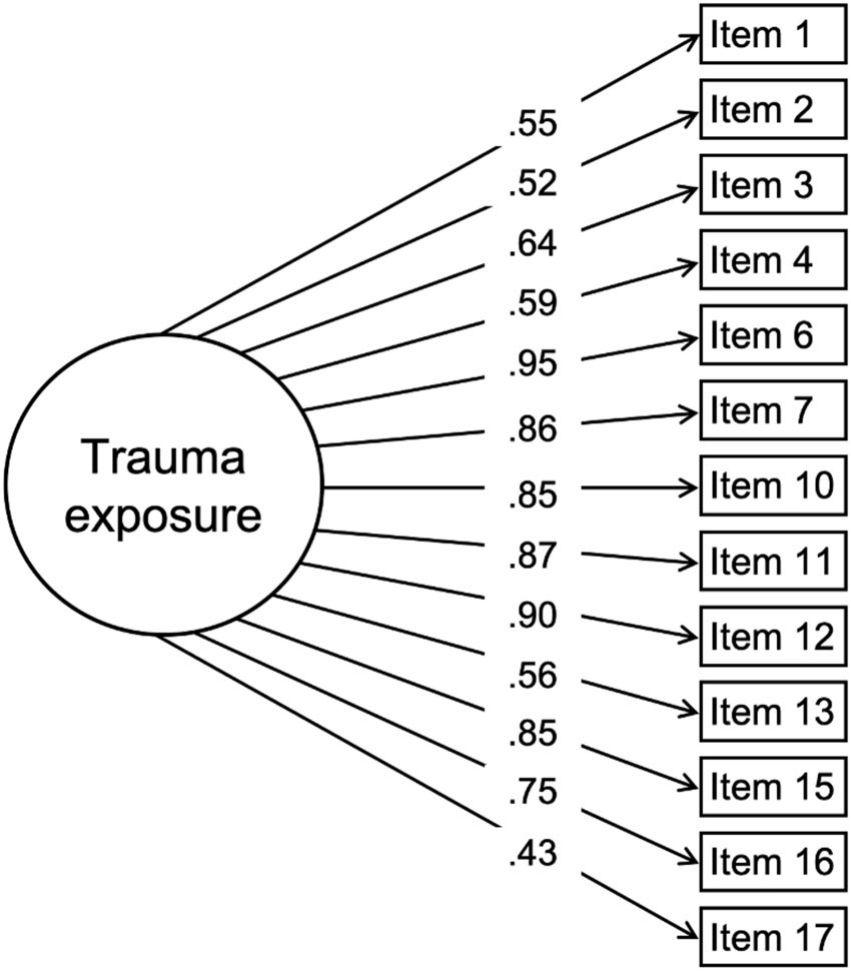
Latent factor of trauma exposure. Trauma exposure was assessed using the traumatic events checklist for posttraumatic stress disorder from the Kiddie Schedule for Affective Disorders and Schizophrenia (K-SADS)^37^. As noted in Table 2, items 5, 8, 9, and 14 were excluded from the factor analysis due to extremely low endorsement. Thus, 13 items from the K-SADS traumatic events checklist were used to derive a latent variable. With trauma items defined dichotomously (yes/no), a unidimensional item-factor analysis^41^ was used to derive a single latent variable, which we called “trauma exposure.” Standardized loadings are shown.

Trauma exposure was assessed by using the posttraumatic stress disorder criterion A traumatic events checklist from the Kiddie Schedule for Affective Disorders and Schizophrenia (K-SADS)^37^ administered to a parent or guardian. The checklist contains 17 items assessing the occurrence of traumatic events (Table 2; see e.g., “A car accident in which your child or another person in the car was hurt bad enough to require medical attention”, “A family member threatened to kill your child”). Factor analysis was used to derive a single latent variable that represents the degree of lifetime trauma exposure to all traumatic events. Prior to factor analysis, four items were eliminated due to extremely low endorsement (Supplement). The scree plot revealed a clear “elbow” after extraction of a single factor (Fig. 1), further supported by the ratio of first to second eigenvalues (7.6) well beyond the traditional cutoff of 3.0^38^. The loadings of the remaining 13 trauma items were used to define trauma exposure (Fig. 2). Correlation coefficients computed for pairwise comparison of the 13 trauma items used to derive a latent trauma factor are presented in Supplementary Fig. 2.

**Table 2:** Traumatic event items from the Kiddie Schedule for Affective Disorders and Schizophrenia (K-SADS).

	K-SADS item
**1**	**A car accident in which your child or another person in the car was hurt bad enough to require medical attention**
**2**	**Another significant accident for which your child needed specialized and intensive medical treatment**
**3**	**Witnessed or caught in a fire that caused significant property damage or personal injury**
**4**	**Witnessed or caught in a natural disaster that caused significant property damage or personal injury**
5	Witnessed or present during an act of terrorism (e.g., Boston marathon bombing)
**6**	**Witnessed death or mass destruction in a war zone**
**7**	**Witnessed someone shot or stabbed in the community**
8	Shot, stabbed, or beaten brutally by a non-family member
9	Shot, stabbed, or beaten brutally by a grown-up in the home
**10**	**Beaten to the point of having bruises by a grown-up in the home**
**11**	**A non-family member threatened to kill your child**
**12**	**A family member threatened to kill your child**
**13**	**Witnessed the grown-ups in the home push, shove or hit one another**
14	A grown-up in the home touched your child in his or her privates, had your child touch their privates, or did other sexual things to your child
**15**	**An adult outside your family touched your child in his or her privates, had your child touch their privates or did other sexual things to your child**
**16**	**A peer forced your child to do something sexually**
**17**	**Learned about the sudden unexpected death of a loved one**

Items in bold were used to derive a latent factor of trauma exposure. Items 5, 8, 9, and 14 were excluded from the factor analysis due to extremely low endorsement (see Supplement).

### Image acquisition, quality assurance, and processing

The description of the image acquisition, quality assurance, and processing procedures for the ABCD Study is detailed elsewhere^39^. A brief summary on procedures developed and performed by the ABCD Data Analysis and Informatics Center (DAIC) and the ABCD Imaging Acquisition Imaging Workgroup is provided in the Supplement.

### Statistical analyses

Structural equation models were conducted in Mplus version 8.4. The mean-adjusted and variance-adjusted weighted least squares (WLSMV) estimator was used with pairwise deletion for missing data^40^. With trauma items defined dichotomously (yes/no), a unidimensional item-factor analysis^41^ was used to derive a single latent variable, which we called “trauma exposure.” Trauma exposure was operationalized using a latent variable created from 13 dichotomous yes/no items about whether the child experienced various traumatic events. The latent factor score represents a weighted sum of these variables, with higher scores indicating exposure to a greater number of traumatic events, while also accounting for measurement error. Post-stratification weights based on propensity scores for age, sex, race/ethnicity, family income, family type and parent employment, household size, and region of the US participants come from, were applied to all analyses to account for the stratification of the sample in data collection sites. Since the ABCD Study includes some participants who are twins or siblings, all analyses took into account clustering within families, with families being modeled with a random intercept.

Analyses were conducted to determine which regions were associated with our latent measure of trauma exposure. Cortical thickness and volume analyses were performed with 68 cortical structures (34 in each hemisphere) based on the Desikan–Killiany atlas^42^. Additionally, volume analyses were performed on 19 subcortical structures^43^. The following demographic factors were included as covariates: age, sex, and race/ethnicity. Additionally, MRI scanner model was included as a covariate to account for differences between scanners. Lastly, average cortical thickness and total cortical and subcortical GMV were included as covariates in cortical thickness and volume analyses, respectively, in order to control for global differences in thickness or volume. Thus, our model was as follows: brain region_i_ = β*age + β*sex + β*race/ethnicity + β*MRI scanner model + β*average cortical thickness or total GMV + β*latent trauma factor, where *i* = 1…68 (i.e., the number of brain regions) for cortical thickness and GMV analysis and *i* = 1…19 for subcortical GMV analysis. The false discovery rate (FDR; *q* < 0.05) was controlled to account for multiple tests across brain regions using the stats package in R version 3.6.1 (http://www.r-project.org/).

### Sensitivity analyses

To test the robustness of our primary findings, income and parent’s highest level of education were added as additional covariates to control for possible associations between low SES and brain structure. For GMV analysis, intracranial volume (ICV) was substituted for total GMV to control for global differences in head size. Additionally, we performed the same analyses using psychopathology factors in place of trauma in order to test whether the structural aberrations we found are specific to trauma or are more broadly related to general psychopathology. For details on how the psychopathology factors were derived, see the Supplement.

## Results

### Trauma exposure is associated with differences in brain structure

**Fig. 3:**
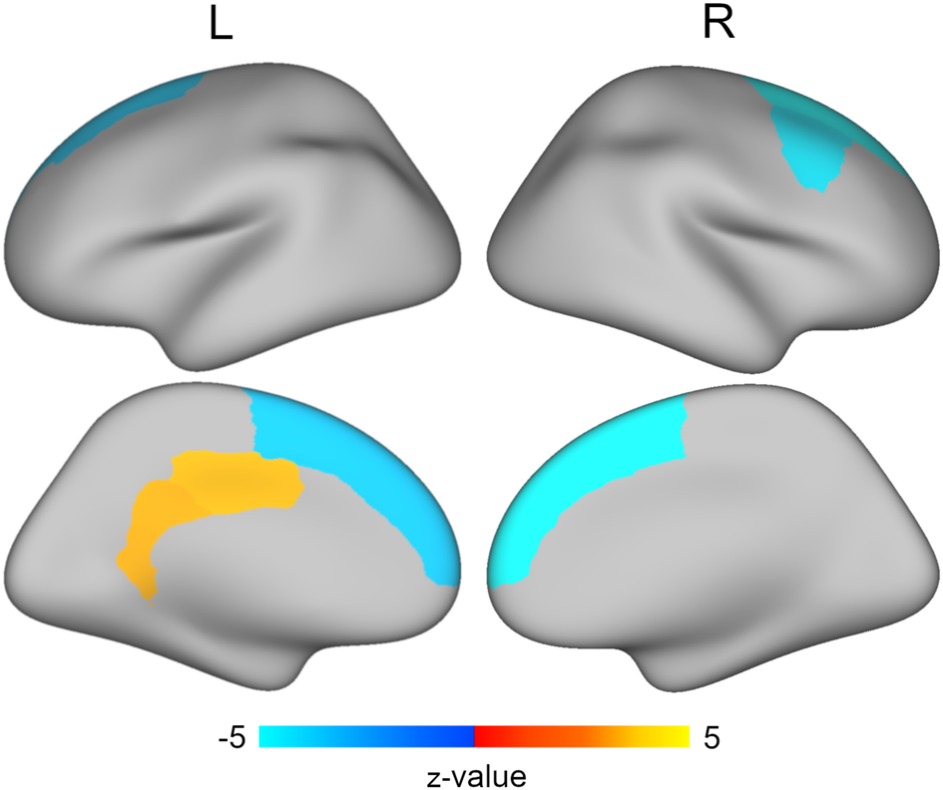
Regions with significant associations between regional cortical thickness and latent trauma. Structural equation modeling that controlled for age, sex, race/ethnicity, scanner model, and average cortical thickness revealed that greater latent trauma scores were associated with thinner cortices in bilateral superior frontal gyri and right caudal middle frontal gyrus (blue) and thicker cortices in left isthmus cingulate and posterior cingulate (yellow). Multiple comparisons were accounted for using the false discovery rate (*q* < 0.05).

After controlling for age, sex, race/ethnicity, scanner model, and average cortical thickness as well as correcting for multiple comparisons, focal results were found in several regions for cortical thickness. Specifically, greater trauma exposure was associated with thinner cortices in bilateral superior frontal gyri and right caudal middle frontal gyrus (Fig. 3 and Supplementary Table 1). Greater trauma exposure was also associated with thicker cortices in the left isthmus cingulate and left posterior cingulate (Fig. 3 and Supplementary Table 1). No other cortical thickness regions were significantly associated with trauma exposure.

In terms of GMV, no cortical regions were significantly associated with trauma exposure after controlling for age, sex, race/ethnicity, scanner model, and total cortical GMV and after correction for multiple comparisons (Supplementary Table 2). For subcortical volume, after controlling for age, sex, race/ethnicity, scanner model, and total subcortical GMV, greater trauma exposure was associated with smaller volumes in the right putamen and the right amygdala (Supplementary Table 3). Of note, there was a weak bilateral effect for these regions: the left putamen and left amygdala were significant at uncorrected levels; however, this did not survive FDR-correction (*p*_fdr_-values = .057). The right hippocampus was also significant at uncorrected levels but did not survive correction (*p*_fdr_ = .057). No other subcortical regions were significantly associated with trauma exposure.

### Sensitivity analyses

Follow-up sensitivity analyses were performed with regional cortical thickness and volume to ensure the primary results were robust to possible confounds. For cortical thickness, the sensitivity findings were largely convergent with the primary results, when controlling for family income and parent’s highest level of education as additional covariates. Greater trauma exposure continued to be negatively associated with bilateral superior frontal gyri and right caudal middle frontal gyrus, and positively associated with the left posterior cingulate (Supplementary Table 4). While trauma exposure was associated with thicker cortices in the left isthmus cingulate at uncorrected levels, this did not survive FDR-correction during sensitivity analyses (*p*_fdr_ = .068). When controlling for family income and parent education for the volume sensitivity analyses, there was no significant association between cortical or subcortical volume and trauma exposure (Supplementary Table 5).

While the volume analyses already controlled for total GMV, it is possible that differences in overall cranial size may be important to consider. Thus, for volume sensitivity analyses, ICV was substituted for total GMV in order to control for cranial size. The cortical volume regions continued to show no significant relationships with trauma exposure when ICV was included as a covariate. In terms of subcortical regions, the right putamen continued to be negatively associated with trauma exposure (Supplementary Table 6). Subcortical regions that were significant at uncorrected levels, but did not survive FDR correction, included the bilateral amygdala and right hippocampus (*p*_fdr_-values ≤ .076).

Lastly, given that trauma exposure is associated with both psychopathology and cortical thickness in this sample, we examined whether our cortical thickness results could be attributed to general psychopathology rather than trauma specifically. To test this, we used psychopathology dimensions derived in our previous work^44^ which defined a general psychopathology factor and three subfactors: internalizing, ADHD, and conduct problems. We then substituted these psychopathology dimensions in place of trauma exposure. These sensitivity analyses showed that greater general psychopathology was associated with thinner cortices in the right paracentral lobule and left postcentral gyrus (Supplementary Table 7). Greater internalizing symptoms were associated with thicker cortices in the right inferior parietal cortex (Supplementary Table 7). Neither ADHD nor conduct problems were significantly associated with cortical thickness differences. When parent’s education and income were controlled, the results with general psychopathology disappeared and the association between internalizing symptoms and the right inferior parietal cortex did not reach significance (*p*_fdr_ = .068; Supplementary Table 8).

## Discussion

The results of the current study, which investigated associations between trauma and brain structure in a large sample of children, demonstrated that trauma exposure was associated with variation in regional cortical thickness and GMV in several key brain regions. Specifically, trauma exposure was associated with thinner cortices in the bilateral superior frontal gyri and right caudal middle frontal gyrus, and with thicker cortices found in the left posterior cingulate and left isthmus cingulate. Additionally, trauma exposure was associated with smaller GMV in the right amygdala and right putamen. Sensitivity analyses revealed that the cortical thickness associations remained largely consistent even when controlling for income and parent education, while no volume results remained significant after controlling for SES. Additionally, when controlling for ICV as an additional covariate for the volume analyses, only the association with the right putamen remained. Finally, results of sensitivity analyses examining associations between psychopathology and cortical thickness yielded significant associations for different regions than the primary analyses focusing on trauma exposure. Overall, this work suggests that trauma exposure during childhood may be a risk factor for structural aberrations in the developing brain.

The findings of thinner frontal cortices in the current study are consistent with prior work^7,11,13,21,45^. The superior frontal gyrus is implicated in working memory and executive functioning^46^, while the middle frontal gyrus is involved in attention modulation and control^47^. Impairments in executive functioning and attention are apparent in individuals with exposure to trauma^48,49^, and high levels of stress have been shown to alter the structure of the prefrontal cortex^50,51^. Animal models demonstrate neuronal reduction in the medial prefrontal cortex due to stress, which is accompanied by functional impairment in working memory performance^52,53^. Relatedly, a meta-analysis demonstrated that early life adversity with threatening components is associated with accelerated maturation of cortical thickness^54^. Frontal regions may be especially susceptible to chronic adversity. Compared to other brain regions, the superior frontal and middle frontal gyri show the greatest age-related reduction in cortical thickness^55^. Such plasticity may increase vulnerability to chronic stressors. Our results support previous findings that trauma exposure is associated with deficits in frontal regions, especially the superior frontal gyrus and caudal middle gyrus.

In contrast to thinner frontal cortices, we found thicker cingulate cortices. This is noteworthy given the prior finding of accelerated decreases in cortical thickness in the posterior and isthmus cortices at faster rates than the global average across ages^56^. The posterior cingulate cortex is highly connected to a broad range of brain regions including frontal, parietal, and subcortical regions and is a key component of the default mode network, which modulates self-referential processing^57,58^. Prior work has shown heightened connectivity within the default mode network in pediatric PTSD patients, which the authors suggest may underlie the persistence of trauma-related memory^59^. Additionally, there is some evidence that thicker cingulate cortices are associated with greater alexithymia (difficulties in identifying, labeling, and communicating one’s emotional state) in patients with PTSD related to childhood maltreatment^60^. However, the analyses in that study were restricted to the dorsal anterior cingulate only. The results of the current study build upon this prior work by demonstrating an association between trauma exposure and thicker posterior cingulate cortices in a large sample of children.

Our volume results are also consistent with prior studies showing that childhood maltreatment is associated with smaller volumes in the amygdala^15,17^ and putamen^61^. The amygdala is central for emotional processing including identifying threatening information^62^, while the putamen is important for motor control and learning^63^. Functional MRI studies show that individuals with trauma exposure demonstrate heightened amygdala activity to threatening stimuli and increased putamen activity to physical pain^9,64,65^. The smaller volumes found in these regions may be the result of accelerated maturation as a result of chronic adversity. Early maltreatment has been suggested to affect the maturation of the brain by increasing normative development-related neuronal atrophy^1,55,66^, thus smaller volumes may result from accelerated pruning. In contrast, it is possible that smaller volumes are the result of insufficient development. This is supported by the finding of slower growth of the amygdala in individuals with a history of maltreatment^19,61^. However, the majority of this work has focused on the broader concept of maltreatment. Our results extend prior work by showing that trauma exposure specifically is associated with structural deficits in the amygdala and putamen.

Of note, in a sensitivity analysis we found that trauma exposure and psychopathology more broadly produced divergent regional cortical thickness results. We found that a general psychopathology factor, also referred to as the *p* factor^67^, was associated with thinner cortices in the paracentral lobule and postcentral gyrus. The paracentral lobule and postcentral gyrus are part of the sensorimotor network and thinner cortices in these regions have been observed in conduct disorder, ADHD, and schizophrenia^68–70^. The divergence between trauma exposure and psychopathology in terms of brain structure is not surprising, as trauma exposure and psychopathology are not equivalent. Many individuals who are exposed to trauma will not develop psychopathology and not all those with psychopathology symptoms were traumatized. Such individual differences may be related to a number of factors, such as resilience or genetics^51,71^. Additionally, although trauma exposure may be a precursor to later psychopathology in some individuals, different neurobiological signatures may be apparent at the time of exposure (e.g., childhood in the current study) compared to the time when symptoms develop (e.g., often later in adolescence^19,72,73^). Importantly, trauma exposure may be related more to internalizing symptoms than general psychopathology. For the specific factor of internalizing symptoms, we found thicker cortices in the inferior parietal cortex. The inferior parietal cortex is associated with the default mode network^58^, similar to our findings with trauma exposure and this same network.

One important limitation of the current study is the use of parental ratings in our measurement of trauma exposure instead of child self-report. Low parental endorsement of items in which a family member could have been a perpetrator of sexual or physical abuse may raise concerns of underreporting of those items. However, while studies indicate that agreement between caregiver-reports and child-reports on prior trauma exposure is poor to moderate^74^, some of the strongest agreements between child and caregiver report included whether the child had been a victim of sexual abuse. At the same time, some of the lowest agreements occurred for physical assaults, with children reporting more violence than caregivers. Thus, the results of the current study may underestimate the impact of certain types of trauma on brain development. Although the current study is limited by the use of parental reports and a cross-sectional design, it has several strengths, including the use of a large sample of children with a precise age range, which is in contrast to the broad age ranges used in many previous cross-sectional studies. Additionally, we examined both GMV and cortical thickness measures in the same sample with rigorous controls for SES. Lastly, the current study focused on the specific construct of trauma, which can be distinguished from the broad conceptualization of childhood maltreatment, using a latent variable of trauma exposure. Future work would benefit from investigating structural differences in distinct types of trauma exposure. For example, childhood sexual abuse, emotional abuse, and peer victimization each show unique structural deficits^10,72^. Although the factor analysis results in the current study suggest that a single trauma exposure construct fits the data well, there may be subfactors of childhood adversity that could be identified using hierarchical modeling in future studies.

In sum, consistent with prior research showing an association between childhood maltreatment and structural brain changes, the present findings suggest that exposure to trauma during childhood is associated with differences in cortical thickness and volume in key regions associated with attention/executive functioning, emotion regulation, and self-referential processing. Thus, childhood trauma exposure may be a risk factor for structural aberrations in the developing brain, which may have implications for the manifestation of psychopathology symptoms later in life. Future waves of the longitudinal ABCD Study dataset will be invaluable to test this hypothesis. The current study provides a pre-pubertal measure before the potential onset of more severe symptoms during adolescence. Additionally, the current study can provide a baseline for establishing the temporal precedence between trauma and structural changes by comparing these characteristics following repeated trauma versus new onset of trauma. This would be a major step towards drawing causal inferences about the relationship between trauma exposure and brain structure, with the hope of informing early interventions for traumatized children.

## Supplementary information

Supplementary Material

## Data Availability

The data from the ABCD Study can be accessed at https://nda.nih.gov/abcd.

## Code availability

The code and corresponding wiki on the analytic procedures are available at https://github.com/VU-BRAINS-lab/Jeong_Trauma_CT.
