# Supplementary material for: The association between latent trauma and brain structure in children
Source: Transl Psychiatry. 2021 Apr 24;11:240. doi: 10.1038/s41398-021-01357-z (PMC8068725; doi:10.1038/s41398-021-01357-z)
Supplement: Supplementary file 1 — Supplementary Material [file 41398_2021_1357_MOESM1_ESM.docx]

**SUPPLEMENTARY MATERIALS**

Table of Contents

[Image Acquisition, Quality Assurance, and Processing 2](#_Toc51592424)

[Factor Analysis 3](#_Toc51592425)

[Psychopathology Analyses 4](#_Toc51592426)

[References 5](#_Toc51592427)

[Supplementary Figure 1. Flowchart indicating exclusions for primary analyses with brain structure and trauma exposure. 6](#_Toc51592428)

[Supplementary Figure 2. Correlation matrix of trauma items. 8](#_Toc51592429)

[Supplementary Table 1. Results examining the relationship between regional cortical thickness and trauma exposure 9](#_Toc51592430)

[Supplementary Table 2. Results examining the relationship between cortical regional GMV and trauma exposure 11](#_Toc51592431)

[Supplementary Table 3. Results examining the relationship between subcortical regional GMV and trauma exposure 13](#_Toc51592432)

[Supplementary Table 4. Results examining the relationship between regional cortical thickness and trauma exposure with income and parent education as additional covariates 14](#_Toc51592433)

[Supplementary Table 5. Results examining the relationship between subcortical regional GMV and trauma exposure with income and parent education as additional covariates 16](#_Toc51592434)

[Supplementary Table 6. Results examining the relationship between subcortical regional GMV and trauma exposure with intracranial volume as a covariate 17](#_Toc51592435)

[Supplementary Table 7. Results of simultaneous regressions examining the relationship between regional cortical thickness and psychopathology dimensions 18](#_Toc51592436)

[Supplementary Table 8. Results of simultaneous regressions examining the relationship between regional cortical thickness and psychopathology dimensions with income and parent education as additional covariates 21](#_Toc51592437)

# **Image Acquisition, Quality Assurance, and Processing**

The ABCD Data Analysis and Informatics Center (DAIC) and the ABCD Imaging Acquisition Workgroup developed an imaging protocol to harmonize collection across multiple 3 tesla (3T) scanner platforms (Siemens Prisma, General Electric (GE) 750, and Phillips) across 21 data collection sites^1^. 3D T1- and T2-weighted images of brain structure were collected. Whole brain T1-weighted images were acquired with the following parameters: TR (repetition time) 2400 to 2500 ms; TE (echo time) 2 to 2.9 ms; FOV (field of view) 256 × 240 to 256; FOV phase of 93.75% to 100%; matrix 256 × 256; 176 to 225 slices; TI (inversion delay) 1060 ms; flip angle of 8°; voxel resolution of 1×1×1×mm; total acquisition time was 7 minutes and 12 seconds for Siemens Prisma, 6 minutes and 9 seconds for GE 750, and 5 minutes and 38 seconds for Phillips.

For imaging data processing and analysis, DAIC used the Multi-Modal Processing Stream (MMPS), a software package developed and maintained at the Center for Multimodal Imaging and Genetic (CMIG) at the University of California, San Diego (UCSD) to employ centralized processing and analysis. Preprocessing was performed which included correction for gradient nonlinearity distortions, intensity scaling and homogeneity correction, registration to an averaged reference brain in standard space, and manual quality control (QC). Then, cortical surface reconstruction and subcortical segmentation were performed based on automated, atlas-based, segmentation procedures in FreeSurfer v.5.3. Next, the morphometric measures were derived by calculating average cortical thickness and average volume in each cortical parcel of the standard FreeSurfer Desikan-Killiany parcellation scheme^2^ and the average volume in each subcortical region^3^. Finally, post-processing QC was performed by trained technicians for motion, intensity homogeneity, white matter underestimation, pial overestimation, and magnetic susceptibility artifact (Supplementary Figure 1). All processing of imaging data was performed by DAIC.

# **Factor Analysis**

We used a pre-existing dataset of 9 270 children, which has sufficient power to detect a small to medium effect size. The data meet the assumptions for all tests performed. A factor analysis was conducted in Mplus version 8.4 to derive a latent factor of trauma exposure that represents the degree of exposure to traumatic events using the items from the trauma checklist from the Kiddie Schedule for Affective Disorders and Schizophrenia (K-SADS)^4^. Items that had low endorsement were eliminated based on the following criteria: 1) traumatic events that were endorsed by less than .5% of the sample, or 2) traumatic events that were endorsed by less than 1% of the sample AND it was not possible to obtain polychoric correlations with other items (contingency tables had blank cells). As a result, four items were eliminated based on these criteria leaving 13 items to define a latent trauma exposure variable. The correlation matrix of trauma items is presented in Supplementary Figure 2. A unidimensional item-factor analysis^5^ was performed to derive a single latent variable, which we defined as “trauma exposure.” The scree plot revealed a clear “elbow” after extraction of a single factor, further supported by the ratio of first to second eigenvalues (7.6) well beyond the traditional cutoff of 3.0^6^.

# **Psychopathology Analyses**

Given that trauma exposure is associated with both psychopathology and cortical thickness in this sample, we examined whether our cortical thickness results could be attributed to general psychopathology rather than trauma specifically. To test this, we used psychopathology dimensions derived in our previous work^7^ which defined a general psychopathology factor and three subfactors. Psychopathology factors were quantified using 66 items from Child Behavior Checklist (CBCL)^8^ based on parent ratings. As previously reported^7^, an exploratory factor analysis clustered the CBCL psychopathological symptoms into three factors: internalizing, ADHD, and conduct problems. Then a confirmatory bifactor analysis was used to model these three specific factors, plus a general psychopathology factor, which represents the symptoms shared across all domains. General psychopathology and the specific psychopathology factors are orthogonal, and thus can be included together in the same model. For additional details on the bifactor model, see Moore et al.^7^. In the current study, we examined the associations between regional cortical thickness and these psychopathological factors. The model for analyses was as follows: region = β*age + β*sex + β*race/ethnicity + β*MRI scanner model + β*average cortical thickness + β*general psychopathology + β*conduct problems + β*internalizing + β*ADHD. The correlation coefficients between the trauma factor and the psychopathology factors were as follows: general psychopathology, *r*(9268) = .21, *p* < .001; conduct problems, *r*(9268) = .21, *p* < .001; internalizing, *r*(9268) = .08, *p* < .001; ADHD, *r*(9268) = .04, *p* < .001.

# **References**

1 Casey BJ *et al.* The Adolescent Brain Cognitive Development (ABCD) study: Imaging acquisition across 21 sites. *Dev. Cogn. Neurosci.*; **32**: 43–54, (2018).

2 Desikan RS *et al.* An automated labeling system for subdividing the human cerebral cortex on MRI scans into gyral based regions of interest. *Neuroimage*; **31**: 968–980, (2006).

3 Fischl B *et al.* Whole brain segmentation: Automated labeling of neuroanatomical structures in the human brain. *Neuron*; **33**: 341–355, (2002).

4 Kaufman J *et al.* Schedule for affective disorders and schizophrenia for school-age children-present and lifetime version (K-SADS-PL): Initial reliability and validity data. *J. Am. Acad. Child Adolesc. Psychiatry*; **36**: 980–988, (1997).

5 Wirth RJ, Edwards MC. Item factor analysis: Current approaches and future directions. *Psychol. Methods*; **12**: 58–79, (2007).

6 Reise SP, Moore TM, Haviland MG. in *APA handbook of testing and assessment in psychology, Vol. 1. Test theory and testing and assessment in industrial and organizational psychology*. (eds Geisinger KF *et al*.) Applying unidimensional item response theory models to psychological data. (American Psychological Association, Wasington, DC, 2013).

7 Moore TM *et al.* Criterion validity and relationships between alternative hierarchical dimensional models of general and specific psychopathology. *J. Abnorm. Psychol.*; **129**: 677–688, (2020).

8 Achenbach TM. *The Achenbach system of empirically based assessment (ASEBA): Development, findings, theory, and applications.* (University of Vermont Research Center for Children, Youth, and Families, Burlington, VT, 2009).


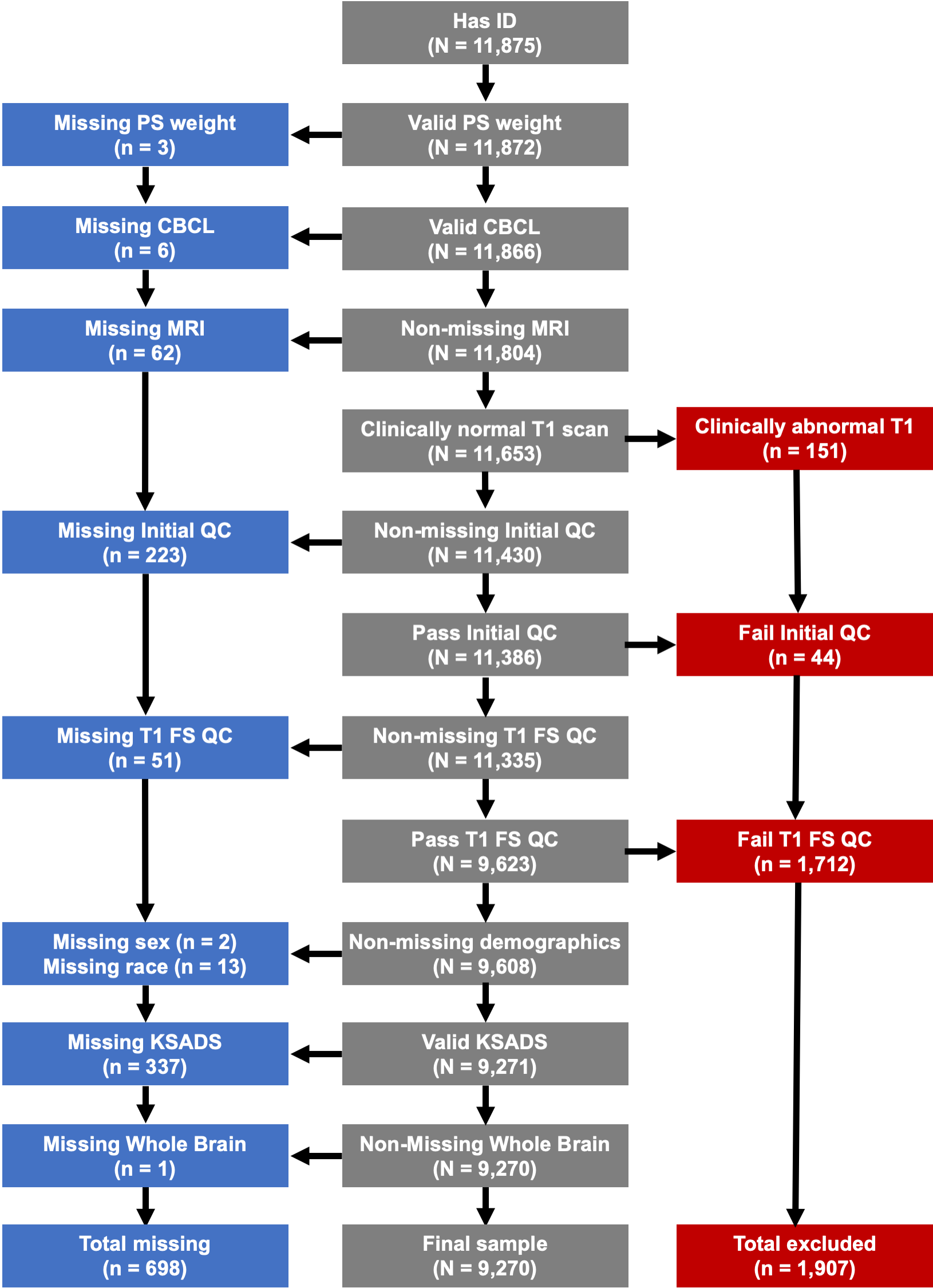


**Supplementary Figure 1. Flowchart indicating exclusions for primary analyses with brain structure and trauma exposure.** Missing: There were 3 participants excluded for missing propensity weight data (PS weight), 6 for missing Child Behavior Checklist data (CBCL), 62 for missing a variable indicating the normality/abnormality of the structural MRI images (“mrif_score”), 223 for missing data on an initial quality assurance variable (“iqc_t1_ok_ser”), 51 for missing data on an additional quality assurance variable (“fsqc_qc”), 2 for missing sex data, 13 for missing race-ethnicity data, 337 for missing items on the K-SADS trauma events checklist, and 1 for missing the whole brain variable (average cortical thickness or total gray matter volume). Exclusion: There were 151 participants excluded for abnormal structural images, as indicated by an “mrif_score” value of 0 (“Image artifacts prevent radiology read”) or 4 (“Consider immediate clinical referral”). There were 44 excluded for failing to pass initial quality control (QC) measures, as indicated by an “iqc_t1_ok_ser” value of 0. There were 1 712 excluded for failing to pass quality assurance variables based on FreeSurfer (FS) QC measures. Specifically, for QC score (“fsqc_qc”), responses of 0 (“reject”) were excluded. For motion score (“fsqc_qu_motion”), pial overestimation score (“fsqc_qu_pialover”), white matter underestimation score (“fsqc_qu_wmunder”), and inhomogeneity (fsqc_qu_inhomogeneity), responses of >1 (“mild” to “severe”) were excluded and only responses of 0 (“absent”) were included.


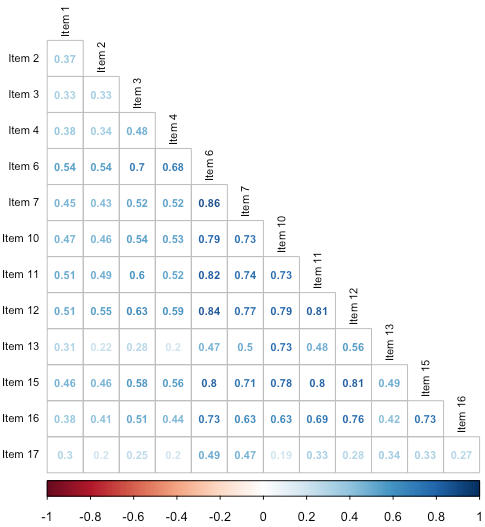


**Supplementary Figure 2. Correlation matrix of trauma items.** Pearson correlation coefficients were computed for pairwise comparison of 13 trauma items used to derive a latent trauma factor.

# **Supplementary Table 1.** *Results examining the relationship between regional cortical thickness and trauma exposure*

| Brain region | Trauma | |  | Brain region | Trauma | |  |
| --- | --- | --- | --- | --- | --- | --- | --- |
|  | *β* | *p_fdr_* | *R^2^* |  | *β* | *p_fdr_* | *R^2^* |
| Left banks of superior temporal sulcus | -0.03 | .306 | .001 | Right banks of superior temporal sulcus | 0.01 | .933 | .000 |
| Left caudal anterior cingulate | 0.03 | .337 | .001 | Right caudal anterior cingulate | -0.01 | .933 | .000 |
| Left caudal middle frontal | -0.04 | .085 | .001 | Right caudal middle frontal | **-0.07** | **<.001** | .005 |
| Left cuneus | 0.00 | .949 | .000 | Right cuneus | -0.01 | .824 | .000 |
| Left entorhinal | 0.03 | .263 | .001 | Right entorhinal | 0.02 | .605 | .000 |
| Left fusiform | 0.01 | .824 | .000 | Right fusiform | -0.01 | .726 | .000 |
| Left inferior parietal | -0.02 | .496 | .000 | Right inferior parietal | 0.00 | .949 | .000 |
| Left inferior temporal | -0.01 | .839 | .000 | Right inferior temporal | -0.01 | .800 | .000 |
| Left isthmus cingulate | **0.06** | **.027** | .004 | Right isthmus cingulate | 0.03 | .263 | .001 |
| Left lateral occipital | 0.00 | .951 | .000 | Right lateral occipital | -0.02 | .334 | .000 |
| Left lateral orbitofrontal | 0.03 | .334 | .001 | Right lateral orbitofrontal | 0.00 | .949 | .000 |
| Left lingual | 0.01 | .800 | .000 | Right lingual | -0.02 | .538 | .000 |
| Left medial orbitofrontal | 0.00 | .940 | .000 | Right medial orbitofrontal | -0.01 | .933 | .000 |
| Left middle temporal | 0.01 | .933 | .000 | Right middle temporal | 0.01 | .696 | .000 |
| Left parahippocampal | 0.03 | .494 | .001 | Right parahippocampal | 0.03 | .495 | .001 |
| Left paracentral | 0.00 | .949 | .000 | Right paracentral | -0.01 | .839 | .000 |
| Left pars opercularis | 0.00 | .949 | .000 | Right pars opercularis | 0.03 | .334 | .001 |
| Left pars orbitalis | 0.01 | .933 | .000 | Right pars orbitalis | 0.02 | .572 | .000 |
| Left pars triangularis | 0.00 | .994 | .000 | Right pars triangularis | 0.00 | .933 | .000 |
| Left pericalcarine | 0.00 | .994 | .000 | Right pericalcarine | -0.02 | .495 | .000 |
| Left postcentral | -0.04 | .085 | .001 | Right postcentral | -0.02 | .572 | .000 |
| Left posterior cingulate | **0.06** | **.017** | .004 | Right posterior cingulate | 0.04 | .263 | .001 |
| Left precentral | -0.02 | .334 | .000 | Right precentral | -0.04 | .143 | .001 |
| Left precuneus | 0.02 | .419 | .001 | Right precuneus | 0.03 | .263 | .001 |
| Left rostral anterior cingulate | -0.02 | .499 | .001 | Right rostral anterior cingulate | 0.01 | .933 | .000 |
| Left rostral middle frontal | -0.02 | .334 | .000 | Right rostral middle frontal | -0.03 | .143 | .001 |
| Left superior frontal | **-0.04** | **<.001** | .002 | Right superior frontal | **-0.06** | **< .001** | .004 |
| Left superior parietal | -0.01 | .933 | .000 | Right superior parietal | -0.01 | .800 | .000 |
| Left superior temporal | -0.02 | .495 | .000 | Right superior temporal | -0.02 | .498 | .000 |
| Left supramarginal | -0.01 | .933 | .000 | Right supramarginal | -0.03 | .185 | .001 |
| Left frontal pole | -0.02 | .538 | .000 | Right frontal pole | -0.01 | .864 | .000 |
| Left temporal pole | 0.03 | .368 | .001 | Right temporal pole | 0.00 | .949 | .000 |
| Left transverse temporal | 0.00 | .949 | .000 | Right transverse temporal | -0.05 | .068 | .002 |
| Left insula | 0.03 | .334 | .001 | Right insula | 0.02 | .538 | .000 |

*Note.* N = 9 270. Coefficients in bold are significant after FDR correction (adopting a 5% false discovery rate) for 68 tests

# **Supplementary Table 2.** *Results examining the relationship between cortical regional GMV and trauma exposure*

| Brain region | Trauma | |  | Brain region | Trauma | |  |
| --- | --- | --- | --- | --- | --- | --- | --- |
|  | *β* | *p_fdr_* | *R^2^* |  | *β* | *p_fdr_* | *R^2^* |
| Left banks of superior temporal sulcus | 0.01 | .818 | .000 | Right banks of superior temporal sulcus | -0.01 | .878 | .000 |
| Left caudal anterior cingulate | 0.02 | .803 | .000 | Right caudal anterior cingulate | -0.05 | .204 | .003 |
| Left caudal middle frontal | -0.01 | .837 | .000 | Right caudal middle frontal | 0.00 | .943 | .000 |
| Left cuneus | -0.01 | .878 | .000 | Right cuneus | 0.01 | .878 | .000 |
| Left entorhinal | 0.03 | .782 | .001 | Right entorhinal | 0.03 | .763 | .001 |
| Left fusiform | -0.01 | .800 | .000 | Right fusiform | 0.04 | .204 | .002 |
| Left inferior parietal | 0.01 | .837 | .000 | Right inferior parietal | 0.02 | .800 | .000 |
| Left inferior temporal | -0.01 | .837 | .000 | Right inferior temporal | -0.01 | .782 | .000 |
| Left isthmus cingulate | 0.01 | .878 | .000 | Right isthmus cingulate | 0.04 | .550 | .001 |
| Left lateral occipital | -0.01 | .837 | .000 | Right lateral occipital | -0.02 | .782 | .000 |
| Left lateral orbitofrontal | 0.03 | .258 | .001 | Right lateral orbitofrontal | 0.02 | .550 | .000 |
| Left lingual | 0.00 | .927 | .000 | Right lingual | -0.01 | .837 | .000 |
| Left medial orbitofrontal | 0.04 | .204 | .002 | Right medial orbitofrontal | 0.00 | .957 | .000 |
| Left middle temporal | -0.01 | .811 | .000 | Right middle temporal | -0.02 | .550 | .001 |
| Left parahippocampal | -0.01 | .811 | .000 | Right parahippocampal | -0.03 | .550 | .001 |
| Left paracentral | 0.04 | .258 | .002 | Right paracentral | -0.01 | .878 | .000 |
| Left pars opercularis | 0.02 | .782 | .001 | Right pars opercularis | 0.02 | .782 | .000 |
| Left pars orbitalis | 0.01 | .803 | .000 | Right pars orbitalis | 0.01 | .837 | .000 |
| Left pars triangularis | -0.01 | .837 | .000 | Right pars triangularis | -0.01 | .837 | .000 |
| Left pericalcarine | 0.00 | .957 | .000 | Right pericalcarine | 0.00 | .975 | .000 |
| Left postcentral | -0.02 | .782 | .000 | Right postcentral | 0.01 | .811 | .000 |
| Left posterior cingulate | 0.01 | .837 | .000 | Right posterior cingulate | 0.00 | .943 | .000 |
| Left precentral | 0.01 | .803 | .000 | Right precentral | -0.03 | .550 | .001 |
| Left precuneus | 0.00 | .957 | .000 | Right precuneus | -0.02 | .782 | .000 |
| Left rostral anterior cingulate | 0.02 | .763 | .000 | Right rostral anterior cingulate | 0.02 | .782 | .000 |
| Left rostral middle frontal | -0.01 | .782 | .000 | Right rostral middle frontal | 0.01 | .800 | .000 |
| Left superior frontal | 0.00 | .943 | .000 | Right superior frontal | 0.02 | .550 | .000 |
| Left superior parietal | 0.02 | .782 | .000 | Right superior parietal | -0.01 | .878 | .000 |
| Left superior temporal | -0.02 | .782 | .000 | Right superior temporal | -0.01 | .837 | .000 |
| Left supramarginal | -0.01 | .800 | .000 | Right supramarginal | -0.02 | .782 | .000 |
| Left frontal pole | -0.01 | .833 | .000 | Right frontal pole | 0.00 | .975 | .000 |
| Left temporal pole | -0.01 | .878 | .000 | Right temporal pole | 0.02 | .800 | .000 |
| Left transverse temporal | -0.02 | .782 | .000 | Right transverse temporal | -0.03 | .763 | .001 |
| Left insula | 0.00 | .943 | .000 | Right insula | 0.02 | .782 | .000 |

*Note.* N = 9 270

# **Supplementary Table 3.** *Results examining the relationship between subcortical regional GMV and trauma exposure*

| Brain region | Trauma | |  | Brain region | Trauma | |  |
| --- | --- | --- | --- | --- | --- | --- | --- |
|  | *β* | *p_fdr_* | *R^2^* |  | *β* | *p_fdr_* | *R^2^* |
| Left cerebellum cortex | 0.02 | .092 | .000 | Right cerebellum cortex | 0.01 | .283 | .000 |
| Left thalamus proper | -0.02 | .480 | .000 | Right thalamus proper | -0.02 | .278 | .001 |
| Left caudate | 0.00 | .914 | .000 | Right caudate | 0.01 | .707 | .000 |
| Left putamen | -0.04 | .057 | .001 | Right putamen | **-0.05** | **.019** | .002 |
| Left pallidum | -0.02 | .480 | .000 | Right pallidum | 0.00 | .914 | .000 |
| Left hippocampus | -0.04 | .092 | .002 | Right hippocampus | -0.04 | .057 | .002 |
| Left amygdala | -0.05 | .057 | .002 | Right amygdala | **-0.05** | **.048** | .002 |
| Left accumbens area | -0.01 | .707 | .000 | Right accumbens area | -0.01 | .770 | .000 |
| Left ventral diencephalon | -0.01 | .629 | .000 | Right ventral diencephalon | 0.00 | .953 | .000 |
| Brain stem | 0.00 | .914 | .000 |  |  |  |  |

*Note.* N = 9 270. Coefficients in bold are significant after FDR correction (adopting a 5% false discovery rate) for 19 tests.

# **Supplementary Table 4**. *Results examining the relationship between regional cortical thickness and trauma exposure with income and parent education as additional covariates*

| Brain region | Trauma | |  | Brain region | Trauma | |  |
| --- | --- | --- | --- | --- | --- | --- | --- |
|  | *β* | *p_fdr_* | *R^2^* |  | *β* | *p_fdr_* | *R^2^* |
| Left banks of superior temporal sulcus | -0.03 | .245 | .001 | Right banks of superior temporal sulcus | 0.01 | .830 | .000 |
| Left caudal anterior cingulate | 0.03 | .392 | .001 | Right caudal anterior cingulate | -0.02 | .572 | .001 |
| Left caudal middle frontal | -0.03 | .216 | .001 | Right caudal middle frontal | **-0.07** | **< .001** | .005 |
| Left cuneus | 0.01 | .746 | .000 | Right cuneus | 0.00 | .961 | .000 |
| Left entorhinal | 0.03 | .307 | .001 | Right entorhinal | 0.02 | .556 | .000 |
| Left fusiform | 0.01 | .861 | .000 | Right fusiform | -0.01 | .830 | .000 |
| Left inferior parietal | -0.03 | .307 | .001 | Right inferior parietal | -0.01 | .830 | .000 |
| Left inferior temporal | -0.01 | .671 | .000 | Right inferior temporal | -0.02 | .556 | .000 |
| Left isthmus cingulate | 0.06 | .068 | .003 | Right isthmus cingulate | 0.03 | .389 | .001 |
| Left lateral occipital | 0.00 | .961 | .000 | Right lateral occipital | -0.01 | .572 | .000 |
| Left lateral orbitofrontal | 0.04 | .159 | .002 | Right lateral orbitofrontal | 0.00 | .987 | .000 |
| Left lingual | 0.02 | .389 | .001 | Right lingual | -0.01 | .861 | .000 |
| Left medial orbitofrontal | 0.00 | .946 | .000 | Right medial orbitofrontal | -0.01 | .746 | .000 |
| Left middle temporal | 0.00 | .938 | .000 | Right middle temporal | 0.01 | .830 | .000 |
| Left parahippocampal | 0.04 | .307 | .001 | Right parahippocampal | 0.04 | .307 | .001 |
| Left paracentral | 0.00 | .938 | .000 | Right paracentral | -0.01 | .847 | .000 |
| Left pars opercularis | -0.01 | .807 | .000 | Right pars opercularis | 0.02 | .389 | .001 |
| Left pars orbitalis | 0.01 | .912 | .000 | Right pars orbitalis | 0.01 | .861 | .000 |
| Left pars triangularis | -0.01 | .847 | .000 | Right pars triangularis | 0.00 | .946 | .000 |
| Left pericalcarine | 0.00 | .945 | .000 | Right pericalcarine | -0.01 | .840 | .000 |
| Left postcentral | -0.04 | .163 | .001 | Right postcentral | -0.01 | .746 | .000 |
| Left posterior cingulate | **0.06** | **.017** | .004 | Right posterior cingulate | 0.04 | .255 | .001 |
| Left precentral | -0.02 | .406 | .000 | Right precentral | -0.03 | .245 | .001 |
| Left precuneus | 0.02 | .430 | .000 | Right precuneus | 0.03 | .307 | .001 |
| Left rostral anterior cingulate | -0.03 | .307 | .001 | Right rostral anterior cingulate | 0.00 | .961 | .000 |
| Left rostral middle frontal | -0.03 | .245 | .001 | Right rostral middle frontal | -0.03 | .153 | .001 |
| Left superior frontal | **-0.05** | **< .001** | .002 | Right superior frontal | **-0.07** | **< .001** | .004 |
| Left superior parietal | -0.01 | .830 | .000 | Right superior parietal | -0.02 | .389 | .000 |
| Left superior temporal | -0.02 | .556 | .000 | Right superior temporal | -0.01 | .830 | .000 |
| Left supramarginal | -0.02 | .584 | .000 | Right supramarginal | -0.03 | .153 | .001 |
| Left frontal pole | -0.01 | .746 | .000 | Right frontal pole | -0.01 | .746 | .000 |
| Left temporal pole | 0.03 | .389 | .001 | Right temporal pole | 0.01 | .912 | .000 |
| Left transverse temporal | 0.00 | .946 | .000 | Right transverse temporal | -0.04 | .153 | .002 |
| Left insula | 0.04 | .245 | .001 | Right insula | 0.03 | .307 | .001 |

*Note.* N = 8 496. Coefficients in bold are significant after FDR correction (adopting a 5% false discovery rate) for 68 tests.

# **Supplementary Table 5.** *Results examining the relationship between subcortical regional GMV and trauma exposure with income and parent education as additional covariates*

| Brain region | Trauma | |  | Brain region | Trauma | |  |
| --- | --- | --- | --- | --- | --- | --- | --- |
|  | *β* | *p_fdr_* | *R^2^* |  | *β* | *p_fdr_* | *R^2^* |
| Left cerebellum cortex | 0.01 | .502 | .000 | Right cerebellum cortex | 0.00 | 0.813 | .000 |
| Left thalamus proper | -0.01 | .813 | .000 | Right thalamus proper | -0.02 | 0.625 | .000 |
| Left caudate | 0.01 | .813 | .000 | Right caudate | 0.01 | 0.798 | .000 |
| Left putamen | -0.02 | .502 | .000 | Right putamen | -0.04 | 0.152 | .001 |
| Left pallidum | -0.01 | .798 | .000 | Right pallidum | 0.00 | 0.951 | .000 |
| Left hippocampus | -0.03 | .502 | .001 | Right hippocampus | -0.03 | 0.502 | .001 |
| Left amygdala | -0.04 | .215 | .002 | Right amygdala | -0.05 | 0.152 | .002 |
| Left accumbens area | -0.01 | .813 | .000 | Right accumbens area | 0.00 | 0.979 | .000 |
| Left ventral diencephalon | -0.01 | .813 | .000 | Right ventral diencephalon | 0.01 | 0.813 | .000 |
| Brain stem | 0.00 | .950 | .000 |  |  |  |  |

*Note.* N = 8 496.

# **Supplementary Table 6.** *Results examining the relationship between subcortical regional GMV and trauma exposure with intracranial volume as a covariate*

| Brain region | Trauma | |  | Brain region | Trauma | |  |
| --- | --- | --- | --- | --- | --- | --- | --- |
|  | *β* | *p_fdr_* | *R^2^* |  | *β* | *p_fdr_* | *R^2^* |
| Left cerebellum cortex | 0.01 | .774 | .000 | Right cerebellum cortex | 0.01 | .894 | .000 |
| Left thalamus proper | -0.01 | .747 | .000 | Right thalamus proper | -0.02 | .426 | .000 |
| Left caudate | 0.01 | .894 | .000 | Right caudate | 0.01 | .764 | .000 |
| Left putamen | -0.03 | .114 | .001 | Right putamen | **-0.05** | **.019** | .002 |
| Left pallidum | -0.02 | .747 | .000 | Right pallidum | 0.00 | 1.000 | .000 |
| Left hippocampus | -0.04 | .114 | .001 | Right hippocampus | -0.04 | .076 | .002 |
| Left amygdala | -0.05 | .076 | .002 | Right amygdala | -0.05 | .067 | .002 |
| Left accumbens area | -0.01 | .877 | .000 | Right accumbens area | -0.01 | .894 | .000 |
| Left ventral diencephalon | -0.01 | .774 | .000 | Right ventral diencephalon | 0.00 | 1.000 | .000 |
| Brain stem | 0.00 | 1.000 | .000 |  |  |  |  |

*Note.* N = 9 270. Coefficients in bold are significant after FDR correction (adopting a 5% false discovery rate) for 19 tests

# **Supplementary Table 7.** *Results of simultaneous regressions examining the relationship between regional cortical thickness and psychopathology dimensions*

| Brain region | General | |  | Specific Conduct | |  | Specific Internalizing | |  | Specific  ADHD | |  |
| --- | --- | --- | --- | --- | --- | --- | --- | --- | --- | --- | --- | --- |
|  | *β* | *p*_fdr_ | *R^2^* | *β* | *p*_fdr_ | *R^2^* | *β* | *p*_fdr_ | *R^2^* | *β* | *p_f_*_dr_ | *R^2^* |
| Left banks of superior temporal sulcus | 0.00 | .887 | .000 | -0.02 | .841 | .000 | 0.00 | .991 | .000 | 0.01 | .938 | .000 |
| Left caudal anterior cingulate | 0.01 | .824 | .000 | 0.02 | .841 | .000 | 0.00 | .991 | .000 | -0.03 | .571 | .001 |
| Left caudal middle frontal | 0.01 | .808 | .000 | -0.02 | .834 | .000 | 0.00 | .991 | .000 | -0.02 | .729 | .000 |
| Left cuneus | -0.01 | .808 | .000 | -0.03 | .834 | .001 | 0.03 | .653 | .001 | -0.01 | .910 | .000 |
| Left entorhinal | -0.01 | .808 | .000 | 0.04 | .834 | .001 | 0.00 | .996 | .000 | -0.01 | .857 | .000 |
| Left fusiform | 0.00 | .887 | .000 | 0.00 | .975 | .000 | 0.01 | .876 | .000 | 0.01 | .857 | .000 |
| Left inferior parietal | 0.00 | .996 | .000 | 0.00 | .975 | .000 | 0.00 | .991 | .000 | 0.01 | .868 | .000 |
| Left inferior temporal | -0.01 | .859 | .000 | 0.02 | .834 | .000 | 0.01 | .950 | .000 | 0.01 | .857 | .000 |
| Left isthmus cingulate | 0.04 | .175 | .001 | 0.00 | .983 | .000 | -0.03 | .653 | .001 | -0.03 | .571 | .001 |
| Left lateral occipital | -0.01 | .638 | .000 | 0.00 | .983 | .000 | 0.01 | .991 | .000 | 0.02 | .677 | .000 |
| Left lateral orbitofrontal | -0.01 | .859 | .000 | 0.02 | .834 | .000 | -0.01 | .876 | .000 | -0.02 | .625 | .001 |
| Left lingual | -0.01 | .740 | .000 | -0.03 | .834 | .001 | 0.00 | .991 | .000 | -0.01 | .881 | .000 |
| Left medial orbitofrontal | -0.02 | .638 | .000 | 0.01 | .841 | .000 | 0.00 | .991 | .000 | 0.02 | .729 | .000 |
| Left middle temporal | 0.00 | .887 | .000 | 0.01 | .841 | .000 | -0.02 | .696 | .000 | 0.02 | .677 | .000 |
| Left parahippocampal | -0.01 | .808 | .000 | 0.00 | .954 | .000 | 0.02 | .765 | .000 | -0.04 | .567 | .002 |
| Left paracentral | -0.02 | .359 | .000 | -0.02 | .841 | .000 | -0.01 | .843 | .000 | 0.00 | .938 | .000 |
| Left pars opercularis | 0.01 | .808 | .000 | 0.00 | .943 | .000 | -0.01 | .884 | .000 | 0.02 | .729 | .000 |
| Left pars orbitalis | 0.01 | .835 | .000 | 0.03 | .834 | .001 | -0.02 | .653 | .000 | 0.01 | .866 | .000 |
| Left pars triangularis | 0.00 | .893 | .000 | 0.01 | .841 | .000 | 0.00 | .991 | .000 | 0.02 | .677 | .000 |
| Left pericalcarine | -0.01 | .845 | .000 | 0.01 | .900 | .000 | 0.01 | .991 | .000 | 0.00 | .938 | .000 |
| Left postcentral | -0.04 | .034 | .001 | -0.01 | .841 | .000 | 0.00 | .991 | .000 | 0.02 | .625 | .001 |
| Left posterior cingulate | 0.03 | .175 | .001 | -0.02 | .834 | .000 | -0.02 | .653 | .000 | -0.01 | .857 | .000 |
| Left precentral | -0.01 | .638 | .000 | -0.03 | .834 | .001 | 0.00 | .991 | .000 | -0.03 | .571 | .001 |
| Left precuneus | 0.00 | .974 | .000 | -0.02 | .834 | .000 | 0.00 | .991 | .000 | -0.01 | .866 | .000 |
| Left rostral anterior cingulate | 0.03 | .163 | .001 | 0.03 | .834 | .001 | -0.01 | .991 | .000 | -0.03 | .625 | .001 |
| Left rostral middle frontal | 0.00 | .974 | .000 | -0.01 | .841 | .000 | 0.00 | .996 | .000 | 0.01 | .809 | .000 |
| Left superior frontal | 0.01 | .845 | .000 | 0.00 | .975 | .000 | -0.01 | .991 | .000 | 0.01 | .874 | .000 |
| Left superior parietal | -0.01 | .808 | .000 | 0.00 | .983 | .000 | 0.00 | .991 | .000 | 0.01 | .857 | .000 |
| Left superior temporal | 0.01 | .824 | .000 | 0.01 | .841 | .000 | 0.00 | .991 | .000 | 0.02 | .625 | .001 |
| Left supramarginal | 0.00 | .887 | .000 | 0.00 | .964 | .000 | 0.00 | .991 | .000 | 0.00 | .938 | .000 |
| Left frontal pole | 0.03 | .356 | .001 | 0.00 | .999 | .000 | -0.02 | .831 | .000 | 0.00 | .938 | .000 |
| Left temporal pole | 0.01 | .845 | .000 | -0.01 | .873 | .000 | -0.02 | .653 | .001 | -0.02 | .857 | .000 |
| Left transverse temporal | 0.02 | .638 | .000 | -0.01 | .841 | .000 | 0.01 | .991 | .000 | 0.02 | .672 | .001 |
| Left insula | -0.01 | .808 | .000 | -0.01 | .841 | .000 | 0.02 | .765 | .000 | 0.01 | .857 | .000 |
| Right banks of superior temporal sulcus | -0.01 | .845 | .000 | -0.02 | .841 | .000 | 0.00 | .991 | .000 | 0.01 | .866 | .000 |
| Right caudal anterior cingulate | 0.02 | .740 | .000 | 0.01 | .894 | .000 | -0.01 | .934 | .000 | -0.04 | .476 | .002 |
| Right caudal middle frontal | -0.01 | .808 | .000 | -0.01 | .841 | .000 | 0.01 | .884 | .000 | -0.01 | .866 | .000 |
| Right cuneus | -0.02 | .638 | .000 | -0.01 | .841 | .000 | 0.01 | .950 | .000 | -0.01 | .910 | .000 |
| Right entorhinal | 0.01 | .870 | .000 | -0.01 | .841 | .000 | 0.00 | .991 | .000 | -0.02 | .809 | .000 |
| Right fusiform | 0.00 | .887 | .000 | -0.02 | .834 | .001 | 0.02 | .653 | .000 | -0.01 | .857 | .000 |
| Right inferior parietal | 0.00 | .870 | .000 | 0.01 | .841 | .000 | 0.04 | < .001 | .001 | 0.00 | .994 | .000 |
| Right inferior temporal | 0.00 | .887 | .000 | 0.01 | .873 | .000 | 0.02 | .696 | .000 | 0.00 | .974 | .000 |
| Right isthmus cingulate | 0.02 | .740 | .000 | 0.01 | .841 | .000 | 0.00 | .991 | .000 | 0.00 | .974 | .000 |
| Right lateral occipital | -0.01 | .638 | .000 | 0.00 | .943 | .000 | 0.02 | .653 | .000 | 0.01 | .809 | .000 |
| Right lateral orbitofrontal | -0.03 | .227 | .001 | 0.02 | .834 | .000 | 0.02 | .653 | .000 | 0.02 | .814 | .000 |
| Right lingual | -0.02 | .333 | .001 | -0.02 | .841 | .000 | 0.02 | .653 | .000 | 0.03 | .571 | .001 |
| Right medial orbitofrontal | 0.01 | .740 | .000 | 0.00 | .983 | .000 | 0.01 | .991 | .000 | 0.00 | .978 | .000 |
| Right middle temporal | 0.01 | .808 | .000 | 0.04 | .136 | .002 | 0.01 | .822 | .000 | 0.04 | .204 | .001 |
| Right parahippocampal | -0.02 | .740 | .000 | 0.01 | .873 | .000 | 0.01 | .884 | .000 | -0.01 | .938 | .000 |
| Right paracentral | -0.04 | .034 | .002 | -0.01 | .841 | .000 | -0.02 | .653 | .000 | 0.01 | .868 | .000 |
| Right pars opercularis | 0.02 | .638 | .000 | -0.01 | .841 | .000 | 0.02 | .653 | .000 | -0.01 | .910 | .000 |
| Right pars orbitalis | 0.01 | .808 | .000 | 0.02 | .841 | .000 | 0.01 | .991 | .000 | 0.03 | .625 | .001 |
| Right pars triangularis | 0.03 | .113 | .001 | -0.01 | .841 | .000 | 0.01 | .991 | .000 | 0.00 | .974 | .000 |
| Right pericalcarine | -0.01 | .845 | .000 | -0.01 | .900 | .000 | 0.00 | .991 | .000 | -0.02 | .677 | .001 |
| Right postcentral | -0.02 | .356 | .000 | -0.01 | .841 | .000 | 0.00 | .996 | .000 | 0.01 | .841 | .000 |
| Right posterior cingulate | 0.00 | .887 | .000 | -0.02 | .841 | .000 | -0.03 | .653 | .001 | -0.01 | .857 | .000 |
| Right precentral | -0.03 | .136 | .001 | -0.01 | .841 | .000 | 0.02 | .653 | .000 | 0.00 | .938 | .000 |
| Right precuneus | -0.01 | .726 | .000 | 0.01 | .873 | .000 | 0.00 | .991 | .000 | -0.01 | .910 | .000 |
| Right rostral anterior cingulate | 0.02 | .356 | .001 | -0.03 | .834 | .001 | -0.01 | .950 | .000 | -0.03 | .625 | .001 |
| Right rostral middle frontal | 0.01 | .845 | .000 | -0.01 | .900 | .000 | 0.00 | .991 | .000 | 0.02 | .729 | .000 |
| Right superior frontal | 0.00 | .887 | .000 | 0.01 | .841 | .000 | -0.01 | .950 | .000 | 0.00 | .994 | .000 |
| Right superior parietal | -0.01 | .808 | .000 | 0.01 | .841 | .000 | 0.00 | .991 | .000 | 0.00 | .995 | .000 |
| Right superior temporal | 0.01 | .845 | .000 | 0.00 | .900 | .000 | -0.01 | .876 | .000 | 0.03 | .567 | .001 |
| Right supramarginal | 0.01 | .808 | .000 | -0.01 | .841 | .000 | 0.00 | .991 | .000 | -0.01 | .857 | .000 |
| Right frontal pole | 0.00 | .887 | .000 | 0.01 | .841 | .000 | -0.03 | .653 | .001 | 0.00 | .982 | .000 |
| Right temporal pole | 0.00 | .919 | .000 | -0.01 | .841 | .000 | 0.02 | .765 | .000 | 0.03 | .567 | .001 |
| Right transverse temporal | -0.01 | .808 | .000 | 0.02 | .841 | .000 | 0.00 | .991 | .000 | 0.05 | .204 | .002 |
| Right insula | -0.03 | .227 | .001 | -0.01 | .873 | .000 | 0.01 | .950 | .000 | -0.01 | .866 | .000 |

*Note.* N = 9 270. Coefficients in bold are significant after FDR correction (adopting a 5% false discovery rate) for 68 tests

# **Supplementary Table 8.** *Results of simultaneous regressions examining the relationship between regional cortical thickness and psychopathology dimensions with income and parent education as additional covariates*

| Brain region | General | |  | Specific Conduct | |  | Specific Internalizing | |  | Specific  ADHD | |  |
| --- | --- | --- | --- | --- | --- | --- | --- | --- | --- | --- | --- | --- |
|  | *β* | *p*_fdr_ | *R^2^* | *β* | *p*_fdr_ | *R^2^* | *β* | *p*_fdr_ | *R^2^* | *β* | *p_f_*_dr_ | *R^2^* |
| Left banks of superior temporal sulcus | 0.00 | 0.014 | .000 | -0.01 | 0.017 | .000 | 0.00 | 0.014 | .000 | 0.02 | 0.017 | .000 |
| Left caudal anterior cingulate | 0.01 | 0.014 | .000 | 0.01 | 0.018 | .000 | 0.00 | 0.015 | .000 | -0.04 | 0.018 | .002 |
| Left caudal middle frontal | 0.01 | 0.012 | .000 | -0.01 | 0.015 | .000 | 0.00 | 0.012 | .000 | -0.01 | 0.016 | .000 |
| Left cuneus | -0.01 | 0.013 | .000 | -0.03 | 0.016 | .001 | 0.02 | 0.014 | .001 | 0.00 | 0.017 | .000 |
| Left entorhinal | -0.02 | 0.014 | .000 | 0.03 | 0.019 | .001 | 0.00 | 0.015 | .000 | -0.02 | 0.018 | .000 |
| Left fusiform | 0.01 | 0.011 | .000 | 0.00 | 0.014 | .000 | 0.01 | 0.012 | .000 | 0.01 | 0.015 | .000 |
| Left inferior parietal | -0.01 | 0.01 | .000 | -0.01 | 0.012 | .000 | 0.00 | 0.011 | .000 | 0.01 | 0.014 | .000 |
| Left inferior temporal | -0.01 | 0.012 | .000 | 0.02 | 0.015 | .000 | 0.01 | 0.012 | .000 | 0.02 | 0.015 | .000 |
| Left isthmus cingulate | 0.03 | 0.016 | .001 | -0.01 | 0.019 | .000 | -0.03 | 0.016 | .001 | -0.04 | 0.02 | .001 |
| Left lateral occipital | -0.02 | 0.01 | .000 | 0.01 | 0.012 | .000 | 0.00 | 0.01 | .000 | 0.02 | 0.013 | .001 |
| Left lateral orbitofrontal | 0.00 | 0.012 | .000 | 0.02 | 0.015 | .001 | -0.01 | 0.012 | .000 | -0.02 | 0.016 | .000 |
| Left lingual | -0.01 | 0.013 | .000 | -0.03 | 0.016 | .001 | 0.00 | 0.013 | .000 | 0.00 | 0.017 | .000 |
| Left medial orbitofrontal | -0.02 | 0.013 | .000 | 0.01 | 0.017 | .000 | -0.01 | 0.014 | .000 | 0.01 | 0.017 | .000 |
| Left middle temporal | 0.00 | 0.011 | .000 | 0.01 | 0.014 | .000 | -0.01 | 0.011 | .000 | 0.03 | 0.015 | .001 |
| Left parahippocampal | -0.01 | 0.015 | .000 | 0.01 | 0.018 | .000 | 0.02 | 0.015 | .000 | -0.04 | 0.019 | .002 |
| Left paracentral | -0.01 | 0.012 | .000 | -0.02 | 0.014 | .000 | -0.01 | 0.012 | .000 | 0.00 | 0.016 | .000 |
| Left pars opercularis | 0.01 | 0.012 | .000 | 0.02 | 0.014 | .000 | -0.02 | 0.012 | .000 | 0.02 | 0.015 | .001 |
| Left pars orbitalis | 0.01 | 0.013 | .000 | 0.03 | 0.017 | .001 | -0.02 | 0.013 | .000 | 0.01 | 0.017 | .000 |
| Left pars triangularis | -0.01 | 0.012 | .000 | 0.01 | 0.015 | .000 | 0.00 | 0.013 | .000 | 0.02 | 0.016 | .000 |
| Left pericalcarine | 0.00 | 0.014 | .000 | 0.01 | 0.017 | .000 | 0.00 | 0.014 | .000 | 0.01 | 0.018 | .000 |
| Left postcentral | -0.03 | 0.011 | .001 | -0.01 | 0.015 | .000 | -0.01 | 0.012 | .000 | 0.03 | 0.015 | .001 |
| Left posterior cingulate | 0.04 | 0.014 | .002 | -0.02 | 0.017 | .000 | -0.02 | 0.014 | .000 | -0.01 | 0.018 | .000 |
| Left precentral | -0.01 | 0.01 | .000 | -0.02 | 0.013 | .000 | 0.00 | 0.011 | .000 | -0.02 | 0.014 | .000 |
| Left precuneus | 0.01 | 0.01 | 0.000 | -0.03 | 0.013 | .001 | 0.00 | 0.011 | .000 | -0.01 | 0.014 | .000 |
| Left rostral anterior cingulate | 0.03 | 0.014 | .001 | 0.02 | 0.017 | .000 | -0.01 | 0.014 | .000 | -0.03 | 0.018 | .001 |
| Left rostral middle frontal | 0.00 | 0.01 | .000 | -0.01 | 0.012 | .000 | 0.01 | 0.01 | .000 | 0.01 | 0.013 | .000 |
| Left superior frontal | 0.01 | 0.01 | .000 | 0.01 | 0.013 | .000 | 0.00 | 0.01 | .000 | 0.01 | 0.013 | .000 |
| Left superior parietal | 0.00 | 0.011 | .000 | -0.01 | 0.012 | .000 | 0.00 | 0.011 | .000 | 0.01 | 0.014 | .000 |
| Left superior temporal | 0.01 | 0.01 | .000 | 0.02 | 0.014 | .000 | 0.00 | 0.011 | .000 | 0.02 | 0.014 | .001 |
| Left supramarginal | 0.00 | 0.01 | .000 | -0.01 | 0.013 | .000 | 0.00 | 0.01 | .000 | 0.01 | 0.014 | .000 |
| Left frontal pole | 0.04 | 0.015 | .001 | 0.00 | 0.017 | .000 | -0.01 | 0.015 | .000 | -0.01 | 0.018 | .000 |
| Left temporal pole | 0.00 | 0.015 | .000 | -0.01 | 0.018 | .000 | -0.02 | 0.014 | .001 | -0.01 | 0.018 | .000 |
| Left transverse temporal | 0.02 | 0.013 | .001 | 0.00 | 0.017 | .000 | 0.00 | 0.014 | .000 | 0.03 | 0.017 | .001 |
| Left insula | -0.01 | 0.014 | .000 | -0.01 | 0.016 | .000 | 0.02 | 0.014 | .000 | 0.01 | 0.018 | .000 |
| Right banks of superior temporal sulcus | 0.00 | 0.013 | .000 | -0.01 | 0.016 | .000 | 0.00 | 0.014 | .000 | 0.01 | 0.018 | .000 |
| Right caudal anterior cingulate | 0.01 | 0.015 | .000 | 0.00 | 0.019 | .000 | -0.01 | 0.015 | .000 | -0.05 | 0.02 | .002 |
| Right caudal middle frontal | -0.01 | 0.012 | .000 | -0.02 | 0.015 | .000 | 0.01 | 0.013 | .000 | -0.02 | 0.017 | .000 |
| Right cuneus | -0.01 | 0.013 | .000 | -0.01 | 0.016 | .000 | 0.01 | 0.013 | .000 | 0.00 | 0.017 | .000 |
| Right entorhinal | 0.00 | 0.015 | .000 | -0.02 | 0.018 | .000 | -0.01 | 0.015 | .000 | -0.02 | 0.019 | .001 |
| Right fusiform | 0.00 | 0.012 | .000 | -0.02 | 0.014 | .000 | 0.01 | 0.011 | .000 | -0.01 | 0.014 | .000 |
| Right inferior parietal | -0.01 | 0.01 | .000 | -0.01 | 0.012 | .000 | 0.03 | 0.01 | .001 | 0.00 | 0.013 | .000 |
| Right inferior temporal | 0.00 | 0.012 | .000 | 0.00 | 0.014 | .000 | 0.01 | 0.012 | .000 | 0.00 | 0.016 | .000 |
| Right isthmus cingulate | 0.01 | 0.015 | .000 | 0.00 | 0.019 | .000 | 0.01 | 0.015 | .000 | -0.01 | 0.02 | .000 |
| Right lateral occipital | -0.01 | 0.011 | .000 | 0.01 | 0.012 | .000 | 0.02 | 0.011 | .000 | 0.02 | 0.013 | .000 |
| Right lateral orbitofrontal | -0.02 | 0.012 | .001 | 0.02 | 0.014 | .000 | 0.02 | 0.013 | .000 | 0.01 | 0.016 | .000 |
| Right lingual | -0.02 | 0.013 | .000 | -0.01 | 0.016 | .000 | 0.02 | 0.013 | .000 | 0.04 | 0.016 | .001 |
| Right medial orbitofrontal | 0.01 | 0.013 | .000 | -0.01 | 0.017 | .000 | 0.01 | 0.013 | .000 | 0.01 | 0.017 | .000 |
| Right middle temporal | 0.01 | 0.011 | .000 | 0.03 | 0.013 | .001 | 0.01 | 0.011 | .000 | 0.04 | 0.013 | .001 |
| Right parahippocampal | -0.01 | 0.014 | .000 | 0.02 | 0.018 | .000 | 0.01 | 0.015 | .000 | -0.01 | 0.019 | .000 |
| Right paracentral | -0.03 | 0.013 | .001 | -0.01 | 0.015 | .000 | -0.03 | 0.014 | .001 | 0.01 | 0.016 | .000 |
| Right pars opercularis | 0.02 | 0.012 | .000 | -0.01 | 0.015 | .000 | 0.02 | 0.013 | .001 | 0.00 | 0.016 | .000 |
| Right pars orbitalis | 0.00 | 0.013 | .000 | 0.02 | 0.016 | .000 | 0.02 | 0.014 | .000 | 0.03 | 0.017 | .001 |
| Right pars triangularis | 0.03 | 0.012 | .001 | -0.01 | 0.014 | .000 | 0.02 | 0.012 | .000 | 0.01 | 0.015 | .000 |
| Right pericalcarine | -0.01 | 0.014 | .000 | 0.01 | 0.018 | .000 | 0.01 | 0.014 | .000 | -0.01 | 0.018 | .000 |
| Right postcentral | -0.02 | 0.012 | .000 | 0.00 | 0.015 | .000 | 0.00 | 0.013 | .000 | 0.01 | 0.016 | .000 |
| Right posterior cingulate | 0.00 | 0.014 | .000 | -0.01 | 0.018 | .000 | -0.03 | 0.015 | .001 | -0.01 | 0.019 | .000 |
| Right precentral | -0.02 | 0.011 | .001 | -0.02 | 0.015 | .000 | 0.02 | 0.013 | .000 | 0.00 | 0.016 | .000 |
| Right precuneus | -0.01 | 0.011 | .000 | -0.01 | 0.014 | .000 | -0.01 | 0.012 | .000 | -0.01 | 0.016 | .000 |
| Right rostral anterior cingulate | 0.02 | 0.014 | .001 | -0.03 | 0.018 | .001 | -0.01 | 0.014 | .000 | -0.04 | 0.018 | .001 |
| Right rostral middle frontal | 0.00 | 0.01 | .000 | -0.01 | 0.013 | .000 | 0.01 | 0.011 | .000 | 0.02 | 0.014 | .000 |
| Right superior frontal | 0.00 | 0.01 | .000 | 0.01 | 0.013 | .000 | -0.01 | 0.011 | .000 | 0.00 | 0.014 | .000 |
| Right superior parietal | -0.01 | 0.011 | .000 | 0.00 | 0.013 | .000 | -0.01 | 0.011 | .000 | 0.00 | 0.015 | .000 |
| Right superior temporal | 0.01 | 0.01 | .000 | 0.00 | 0.014 | .000 | -0.01 | 0.011 | .000 | 0.03 | 0.014 | .001 |
| Right supramarginal | 0.01 | 0.01 | .000 | -0.02 | 0.013 | .000 | 0.00 | 0.01 | .000 | -0.01 | 0.013 | .000 |
| Right frontal pole | -0.01 | 0.015 | .000 | 0.01 | 0.017 | .000 | -0.02 | 0.015 | .000 | 0.00 | 0.018 | .000 |
| Right temporal pole | 0.00 | 0.014 | .000 | 0.00 | 0.016 | .000 | 0.01 | 0.014 | .000 | 0.03 | 0.017 | .001 |
| Right transverse temporal | -0.01 | 0.014 | .000 | 0.02 | 0.017 | .001 | -0.01 | 0.014 | .000 | 0.05 | 0.017 | .002 |
| Right insula | -0.02 | 0.014 | .000 | 0.00 | 0.017 | .000 | 0.01 | 0.015 | .000 | -0.01 | 0.019 | .000 |

*Note.* N = 9 270. Coefficients in bold are significant after FDR correction (adopting a 5% false discovery rate) for 68 tests
